# Association of Fat Body Mass With Vertebral Fractures in Postmenopausal Women With Early Breast Cancer Undergoing Adjuvant Aromatase Inhibitor Therapy

**DOI:** 10.1001/jamanetworkopen.2019.11080

**Published:** 2019-09-27

**Authors:** Rebecca Pedersini, Vito Amoroso, Filippo Maffezzoni, Fabio Gallo, Antonella Turla, Sara Monteverdi, Mara Ardine, Marco Ravanelli, Lucia Vassalli, Filippo Rodella, Anna Maria Formenti, Alberto Dalla Volta, Edda Lucia Simoncini, Andrea Giustina, Roberto Maroldi, Alfredo Berruti

**Affiliations:** 1 Medical Oncology Unit, Department of Medical and Surgical Specialties, Radiological Sciences and Public Health, University of Brescia, Spedali Civili Hospital, Brescia, Italy; 2 Radiology Unit, Department of Medical and Surgical Specialties, Radiological Sciences and Public Health, University of Brescia, Spedali Civili Hospital, Brescia, Italy; 3 Division of Endocrinology, San Raffaele Vita-Salute University, Milan, Italy; 4 Breast Unit, Spedali Civili Hospital, Brescia, Italy

## Abstract

**Importance:**

Aromatase inhibitors induce a profound depletion in serum estrogen levels. Postmenopausal obese women receiving aromatase inhibitor therapy may be at increased risk of bone fractures owing to the detrimental association of adiposity with bone quality and the loss of the protective effect of estrogens on bone mineral density.

**Objective:**

To determine whether fat body mass (FBM), as measured by dual-energy x-ray absorptiometry, is associated with vertebral fracture prevalence in postmenopausal women undergoing adjuvant aromatase inhibitor therapy for breast cancer.

**Design, Setting, and Participants:**

In this single-center, cross-sectional study, 556 postmenopausal women with early-stage breast cancer were consecutively enrolled from October 15, 2013, to June 30, 2018, and stratified according to whether they were aromatase inhibitor–naive or aromatase inhibitor–treated for at least 2 years. The database was locked on December 31, 2018, and data analysis was completed on February 28, 2019. Eligible patients in both groups had normal renal function, no metabolic diseases, and no previous or current treatment with antiosteoporotic drugs or glucocorticoids. Previous chemotherapy, but not tamoxifen, was permitted. Data were gathered once, at baseline.

**Main Outcomes and Measures:**

Vertebral fracture prevalence associated with FBM in aromatase inhibitor–naive and aromatase inhibitor–treated patients.

**Results:**

Of the 556 women enrolled, the mean age was 63.0 years (95% CI, 62.2-63.8 years). The 195 aromatase inhibitor–treated patients were older than the 361 aromatase inhibitor–naive patients (mean age, 66.1 vs 61.3 years; *P* < .001), had a higher body mass index (mean, 26.4 vs 25.3; *P* = .009), were less likely to engage in physical activity (65.3% vs 73.7%; *P* = .03), and were less likely to consume alcoholic beverages (68.4% vs 80.9%; *P* = .001). Among the aromatase inhibitor–naive patients, the vertebral fracture prevalence was higher in the subgroup with FBM below the median value than in those with high FBM, but the difference was not statistically significant (19.2% vs 13.3%; *P* = .13). Conversely, the proportion of vertebral fractures in the aromatase inhibitor–treated group was 20.0% in patients with low FBM vs 33.3% in patients with high FBM (*P* = .04). An opposite trend in the association of FBM with vertebral fracture prevalence according to aromatase inhibitor group was shown by multivariable analysis in the propensity score–matched sample: odds ratio, 0.38 (95% CI, 0.12-1.19) and 1.94 (95% CI, 0.67-5.64) in the aromatase inhibitor–naive and aromatase inhibitor–treated groups, respectively (odds ratio for the interaction, 5.77 [95% CI, 1.08-30.81]; *P* for interaction term = .03).

**Conclusions and Relevance:**

Fat body mass may be associated with fragility-related fractures in patients with breast cancer who undergo aromatase inhibitor therapy. If these data are confirmed, obesity could be included in the algorithm for assessing fracture risk and selecting patients to receive bone resorption inhibitors.

## Key Points

**Question:**

Is fat body mass associated with the prevalence of vertebral fracture in patients with breast cancer undergoing aromatase inhibitor therapy?

**Findings:**

This cross-sectional study of 556 postmenopausal women with early-stage breast cancer treated with aromatase inhibitors found that high fat body mass was associated with a numerically but not significantly lower proportion of vertebral fractures in aromatase inhibitor–naive women and a significantly higher proportion of vertebral fractures in aromatase inhibitor–treated women. This opposite trend in fracture prevalence was confirmed after propensity-score matching.

**Meaning:**

Fat body mass may be a factor associated with vertebral fractures in postmenopausal women with breast cancer receiving aromatase inhibitors.

## Teaser

This cross-sectional study evaluates whether fat body mass is associated with vertebral fracture prevalence among postmenopausal women undergoing adjuvant aromatase inhibitor therapy for breast cancer.

## Introduction

Aromatase inhibitors (AIs) are the standard adjuvant therapy for postmenopausal patients with endocrine-sensitive early-stage breast cancer.^1^ These drugs induce a profound reduction in serum estrogen, which has been shown to lead to bone loss^2,3,4,5,6,7,8,9^ and a marked increase in bone resorption.^4,10^ As a result, women receiving adjuvant AI therapy for breast cancer are at increased risk for bone fractures^2,4,5,6^ and, consequently, increased morbidity and mortality.^11^ A recent cross-sectional study^12^ observed a 12% absolute increase in the prevalence of vertebral fractures, as measured by a quantitative morphometric approach, in AI-treated patients compared with AI-naive patients. Identifying factors associated with fracture risk is of paramount importance when referring patients for preventive measures. A surrogate parameter of fracture risk in healthy postmenopausal women is bone mineral density (BMD) measurement by dual-energy x-ray absorptiometry (DXA). Current guidelines also recommend the use of BMD as a parameter in the assessment of fracture risk among patients with early breast cancer receiving AI therapy.^13^

Randomized clinical trials have revealed that a large number of clinical fractures occur during AI therapy in patients with breast cancer who are not osteoporotic at baseline.^2^ The Adjuvant Denosumab in Postmenopausal Patients With Hormone Receptor–Positive Breast Cancer (ABCSG-18) clinical trial compared adjuvant denosumab with placebo in postmenopausal women with early breast cancer who were undergoing AI therapy and found no difference in fracture risk in the placebo arm among osteopenic patients compared with patients with normal BMD.^14^ This indicates that BMD measurement may be inadequate to identify patients treated with AI for breast cancer who are at risk of osteoporotic fracture.

Bone strength reflects the integration of bone density and bone quality, which consists of a composite of balanced remodeling, adequate mineralization, and microarchitecture changes. In a previous cross-sectional study,^12^ BMD was significantly associated with vertebral fractures in AI-naive but not AI-treated patients with breast cancer. This difference suggests that the pathogenesis of bone fragility in AI-treated patients may differ from that of the general population of postmenopausal women without a diagnosis of early-stage breast cancer, and that bone quality alterations not detected by BMD measurement may have a more relevant role in patients with AI-treated breast cancer than in postmenopausal women.^15^

Because BMD is relatively normal or even high in the general obese population,^16,17^ obese women (body mass index [calculated as weight in kilograms divided by height in meters squared], ≥30) are believed to be at lower risk for osteoporotic fractures than women with a low body mass index.^18,19,20,21^ Higher estrogen levels in women with obesity who are produced by the increased aromatase activity in adipose tissue may account for this protective effect. Accumulating evidence, however, depicts a complex association between adiposity and osteoporosis.^22^ Obesity is associated with adverse changes in bone health via several mechanisms, including alteration of bone-regulating hormones, increased oxidative stress and inflammation, and altered bone cell metabolism.^23^ Obesity may negatively affect bone quality. A greater risk of bone fracture for a given BMD value has been observed in the obese population.^24^ Consistent with this observation, obese patients receiving AI therapy may be at higher risk for bone fractures owing to the loss of the protection associated with estrogens and the detrimental changes in bone quality associated with adiposity. Although body mass index, an inexpensive and validated measure, has its place in the diagnosis of obesity, body composition, as assessed by DXA, measures excess adiposity more accurately than anthropometry.^25^

We conducted a cross-sectional study at the Medical Oncology Unit and Breast Unit of ASST Spedali Civili of Brescia, University of Brescia, Italy. The study compared the prevalence of and factors associated with vertebral fracture in postmenopausal women with early-stage breast cancer undergoing AI therapy vs postmenopausal women with early-stage breast cancer who were AI naive. The association of BMD and several hormones with vertebral fracture prevalence was initially investigated in a smaller cohort of this study.^12^ Herein, we present the data on the association of fat body mass (FBM) and vertebral fracture prevalence in a larger patient cohort, including patients analyzed in a previously published report.^12^

## Methods

### Study Design and Patient Population

This study followed the Strengthening the Reporting of Observational Studies in Epidemiology (STROBE) reporting guideline.^26^ Participation in this cross-sectional study was offered to consecutive postmenopausal women with hormone receptor–positive early-stage breast cancer who were referred to our institution from October 15, 2013, to June 30, 2018. All participants were assessed 1 time, at baseline. The database was locked on December 31, 2018, and data analysis was completed on February 28, 2019. Five hundred fifty-six women were enrolled in the study, 361 were assessed before initiating adjuvant endocrine therapy (AI-naive group), and 195 were assessed while receiving adjuvant AI therapy (AI-treated group). The ethics committee of Brescia, Italy, approved the study protocol and the informed consent forms according to the tenets of the Declaration of Helsinki.^27^ Participants provided written informed consent.

The primary aim of the study was to assess the relationship between FBM and fracture prevalence in the 2 patient subsets: AI-naive and AI-treated patients with breast cancer. The secondary aims were to determine the correlations of fracture risk factors (eg, BMD, age, history of bone fractures, smoking history, alcohol consumption) with the occurrence of vertebral fracture; and to evaluate the difference in trabecular bone score (TBS) calculated on lumbar spine DXA scans between AI-naive and AI-treated patients and to determine the correlation of TBS with FBM.

Eligible patients met the following inclusion criteria: histologic diagnosis of breast cancer, American Joint Committee on Cancer stages I to III, no bone metastases as assessed by bone scintigraphy, no bone metabolic disease, normal renal function, and no previous or current treatment with antiosteoporotic drugs (except for calcium and vitamin D) or glucocorticoids. Previous chemotherapy was permitted, but tamoxifen use was not. Patients in the AI-treated group entered the study if they had been receiving AI therapy for at least 2 years.

### Assessments

Fat body mass was assessed by DXA (Explorer Hologic Inc), as reported previously.^12,25^ The coefficient of variation was 1.5%. Vertebral fractures were assessed by 2 physicians (F.M. and A.M.F.), who were blinded to patient group assignment, using a quantitative morphometric analysis of DXA images. The fractures were classified as mild (height ratio decrease of 20%-25%), moderate (decrease of 26%-40%), or severe (decrease of ≥41%).^28^ Discordant cases were solved by consensus.

Trabecular bone score measurement was performed using the TBS software installed on the densitometer (Explorer Hologic Inc). The TBS was calculated based on the raw data acquired in the DXA scan, evaluating the same regions of measurement as those used for lumbar spine BMD and without further administration of ionizing radiation to the patient. Trabecular bone score, a continuous variable like BMD, was interpreted using the tertile approach extracted from the fracture data of a large Canadian cohort. Degraded microarchitecture represents the highest risk and is defined as a TBS of 1.2 or lower. Partially degraded microarchitecture is a borderline risk and is defined as a TBS between 1.2 and 1.35, whereas normal microarchitecture is defined as a TBS of 1.35 or greater.^29,30^ In addition, patients completed a structured questionnaire for collecting anthropometric variables (ie, height, weight) and lifestyle factors.

### Statistical Analysis

The difference between means between the AI-naive and AI-treated groups was determined with a nonparametric Mann-Whitney test, and the difference between percentages was determined with the χ^2^ test. The correlation between the continuous variables TBS and FBM was evaluated by calculating the Pearson coefficient.

To obtain a more similar set of participants in each group, propensity score nearest-neighbor matching was performed. Baseline characteristics used to estimate the propensity score were the covariates associated with treatment assignment.^31^ The balanced postmatch analysis was performed using the overall χ^2^ test and the univariate mixed-effects logistic regression models, as well as graphical approaches. After that, the univariate mixed-effects logistic regression models were performed to screen the effect of the clinical and demographic variables on the vertebral fracture prevalence. The prevalence odds ratio (OR) associated with vertebral fracture was calculated with its 95% CI for each factor from the mixed-effects logistic regression model. The likelihood ratio test was used as a test of statistical significance. Covariates with *P* < .10 were selected for the multivariable analysis, in which the vertebral fracture prevalence was the dependent variable. Multivariable analysis was performed using again the mixed-effects logistic regression model, and the model selection was done by the Akaike information criterion.^32^

Multiplicative interaction terms were used to test whether FBM differed according to the AI group. For those results suggestive of an interaction with the FBM factor, a stratified analysis was performed based on the AI group data using the mixed-effects logistic regression model.

The associations between TBS, FBM, and AI group in the propensity score–matched sample were explored using the mixed-effects linear regression model. The multiplicative interaction term was used to test whether FBM was different according to the AI group.

Owing to the exploratory design of this study, no adjustment for multiple testing was performed and a formal sample size was not calculated. Statistical significance was defined as a 2-tailed *P* < .05, and data were acquired and analyzed in R version 3.5.3 software environment (The R Foundation).

## Results

Among the 556 women enrolled, the mean age was 63.0 years (95% CI, 62.2-63.8 years). Table 1 presents the clinical and tumor characteristics of the study population. The 195 AI-treated patients were older than the 361 AI-naive patients (mean age, 66.1 [95% CI, 65.0-67.2] vs 61.3 [95% CI, 60.3-62.4] years; *P* < .001), had a higher body mass index (mean, 26.4 [95% CI, 25.7-27.0] vs 25.3 [95% CI, 24.8-25.8]; *P* = .009), were less likely to engage in physical activity (65.3% vs 73.7%; *P* = .03), and were less likely to consume alcoholic beverages (68.4% vs 80.9%; *P* = .001).

**Table 1.** Clinical and Tumor Characteristics of AI-Naive and AI-Treated Patients With Breast Cancer

Characteristic	No. (%)		*P* Value^a^
	AI-Naive Patients (n = 361)	AI-Treated Patients (n = 195)	
Age, mean (95% CI), y	61.3 (60.3-62.4)	66.1 (65.0-67.2)	<.001
BMI, mean (95% CI)	25.3 (24.8-25.8)	26.4 (25.7-27.0)	.009
Pathologic tumor stage			
pT1	240 (67.0)	138 (71.5)	.53
pT2	105 (29.3)	48 (24.9)	
pT3-4	13 (3.6)	7 (3.6)	
Pathologic nodal stage			
pN0	212 (58.7)	115 (59)	.26
pN1	124 (34.3)	59 (30.3)	
pN2-3	18 (5.0)	18 (9.2)	
Unknown	7 (2.0)	3 (1.5)	
Histologic features			
Invasive carcinoma NOS	283 (78.4)	162 (83.1)	.10
Lobular carcinoma	59 (16.4)	28 (14.4)	
Other	18 (5)	2 (1)	
Ki-67 index, mean (range), %	25 (1-90)	18 (0-98)	<.001
*ERBB2* status^b^			
Negative	281 (77.8)	167 (86.5)	.03
Positive	80 (22.2)	26 (13.5)	
Chemotherapy use			
No	200 (55.4)	138 (71.5)	<.001
Yes	161 (44.6)	55 (28.5)	
Previous fractures			
No	295 (81.7)	141 (72.3)	.002
Yes	55 (15.2)	52 (26.7)	
Unknown	11 (3.0)	2 (1.0)	
Smoking status			
No	60 (17.6)	33 (17.0)	.48
Yes	281 (82.4)	161 (83.0)	
Physical activity			
No	79 (26.3)	67 (34.7)	.03
Yes	221 (73.7)	126 (65.3)	
Alcohol consumption			
No	61 (19.1)	61 (31.6)	.001
Yes	258 (80.9)	132 (68.4)	

Abbreviations: AI, aromatase inhibitor; BMI, body mass index (calculated as weight in kilograms divided by height in meters squared); NOS, not otherwise specified.

^a^ *P* value from the Mann-Whitney test or the χ^2^ test as appropriate.

^b^ Formerly *HER2* or *HER2/neu*.

Mean BMD values did not differ significantly between the AI groups. The lumbar spine *T* score was lower in the AI-treated group (mean *T* score, –1.56; 95% CI, –1.72 to –1.40 vs –1.24; 95% CI, –1.39 to –1.07; *P* = .03), and the mean FBM was higher in the AI-treated group (26 676; 95% CI, 25 402-27 951 vs 24 754; 95% CI, 23 888-25 620 g; *P* = .03) (Table 2).

**Table 2.** Distribution of DXA Absorptiometry Parameters in AI-Naive and AI-Treated Patients With Breast Cancer

Characteristic	Group, No. (%)		*P* Value^a^
	AI-Naive	AI-Treated	
No. of patients	361 (64.9)	195 (35.1)	NA
DXA			
Normal	79 (21.9)	24 (12.3)	.003
Osteopenia/osteoporosis	282 (78.1)	171 (87.7)	
Morphometric vertebral fracture			
Not present	302 (83.7)	142 (72.8)	.002
Present	59 (16.3)	53 (27.2)	
Vertebral fracture grade			
No	302 (83.7)	142 (72.8)	.006^b^
Mild	31 (8.6)	30 (15.4)	
Moderate/severe	28 (7.8)	23 (11.8)	
No. of vertebral fractures			
0	302 (83.7)	142 (72.8)	<.001^b^
1	44 (12.2)	31 (15.9)	
≥2	15 (4.2)	22 (11.3)	
Lumbar spine, mean (95% CI)			
BMD, g/cm^2^	0.897 (0.88 to 0.91)	0.872 (0.85 to 0.88)	.09
*T* score	−1.24 (−1.39 to −1.07)	−1.56 (−1.72 to −1.40)	.03
Femoral neck, mean (95% CI)			
BMD, g/cm^2^	0.69 (0.68 to 0.70)	0.68 (0.67 to 0.69)	.46
*T* score	−1.40 (−1.50 to −1.31)	−1.48 (−1.59 to −1.36)	.49
Total hip, mean (95% CI)			
BMD, g/cm^2^	0.82 (0.81 to 0.83)	0.81 (0.80 to 0.83)	.72
*T* score	−0.99 (−1.09 to −0.90)	−1.07 (−1.18 to −0.96)	.45
Body mass, mean (95% CI), g			
Lean	39 580 (39 059 to 40 100)	39 893 (39 200 to 40 585)	.50
Fat	24 754 (23 888 to 25 620)	26 676 (25 402 to 27 951)	.03
TBS, mean (95% CI)^c^	1.26 (1.24 to 1.29)	1.22 (1.19 to 1.25)	.006

Abbreviations: AI, aromatase inhibitor; BMD, bone mineral density; DXA, dual-energy x-ray; TBS, trabecular bone score.

^a^ *P* value from the Mann-Whitney test or the χ^2^ test as appropriate.

^b^ χ^2^ for trend test.

^c^ The TBS was assessed in a subset of 78 AI-naive and 50 AI-treated patients.

The univariate relationships between the prevalent vertebral fracture and DXA parameters in AI groups considered separately are reported in eTable 1 in the Supplement. Lower femoral neck and total hip BMD and *T*-score values correlated significantly with the occurrence of vertebral fracture compared with no vertebral fracture in the AI-naive patients (mean total hip BMD, 0.77 [95% CI, 0.74-0.79] vs 0.82 [95% CI, 0.81-0.84] g/cm^2^; *P* = .001), but not in the AI-treated patients (mean total hip BMD, 0.82 [95% CI, 0.78-0.84] vs 0.81 [95% CI, 0.79-0.82] g/cm^2^; *P* = .84). The occurrence of vertebral fracture was significantly associated with a higher mean FBM in the AI-treated patients (28 717 g [95% CI, 26 343-31 092 g] vs 25 915 g [95% CI, 24 407-27 422 g]; *P* = .03) but not in the AI-naive patients (23 951 g [95% CI, 21 667-26 234 g] vs 24 911 g [95% CI, 23 972-25 850 g]; *P* = .21).

### Prevalence of and Factors Associated With Vertebral Fracture in AI-Naive and AI-Treated Patients

The prevalence of morphometric vertebral fracture was significantly higher among the AI-treated patients than the AI-naive patients (53 [27.2%] vs 59 [16.3%]; *P* = .002). Also, the proportions of patients with moderate or severe fracture (23 [11.8%] vs 28 [7.8%]; *P* = .006) and multiple fractures (22 [11.3%] vs 15 [4.2%]; *P* < .001) were significantly higher in the AI-treated patients than in the AI-naive patients (Table 2).

**Figure 1.**
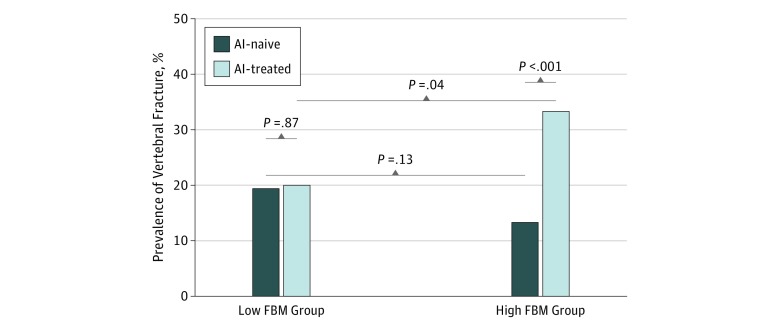
Prevalence of Vertebral Fracture by Fat Body Mass (FBM) Category in Aromatase Inhibitor (AI)–Naive and AI-Treated Patients Low FBM is considered less than the median FBM of participants; high FBM, median or greater.

After dividing patients by FBM categorized at the median value, we noted a numerically higher rate of vertebral fractures in the subset of AI-naive patients with low FBM (less than median) as compared with those with high FBM (median or greater). This difference was not statistically significant (19.2% vs 13.3%; *P* = .13) (Figure 1). Conversely, in the AI-treated group, the proportion of vertebral fractures was lower in the patients with low FBM than in those with high FBM (20.0% vs 33.3%; *P* = .04). Figure 1 shows that the proportion of vertebral fractures among patients with low FBM was similar in the AI-naive (36 of 188) and AI-treated (18 of 90) groups (fracture prevalence, 19.1% vs 20.0%; *P* = .87). On the other hand, the proportion of vertebral fractures in patients with high FBM differed between the AI-treated patients (35 of 105) and AI-naive patients (23 of 173) (fracture prevalence, 33.3% vs 13.3%; *P* < .001).

The quantitative morphometric approach revealed no difference in the proportion of moderate/severe fractures between the AI-naive patients (20 of 180 [11.1%]) and the AI-treated patients (11 of 97 [11.3%]) in the subset of patients with low FBM, whereas it was almost 3 times greater in the AI-treated patients (12 of 98 [12.2%]) than the AI-naive patients (8 of 181 [4.4%]) in the subset with high FBM (eTable 2 in the Supplement). The same trend was observed for the prevalence of mild fractures that was similar in the AI-naive patients (15 of 180 [8.3%]) and the AI-treated patients (9 of 97 [9.3%]) with low FBM, whereas in the high-FBM subset it was almost 2.5 times higher among AI-treated patients (21 of 98 [21.4%]) than AI-naive patients (16 of 181 [8.8%]).

The baseline characteristics of age, previous fractures, chemotherapy use, Ki-67 index, *ERRB2* (formerly *HER2* or *HER2/neu*) status, and alcohol consumption were used in computing the propensity score. Baseline characteristics were significantly associated with the AI group (OR for age, 1.06; 95% CI, 1.04-1.08; *P* < .001; OR for previous fractures, 1.98; 95% CI, 1.29-3.04; *P* = .002) before doing matching. With regard to postmatch analysis, no significant differences were found among baseline characteristics and AI groups (OR for age, 1.00; 95% CI, 0.98-1.03; *P* = .74; OR for previous fractures, 1.26; 95% CI, 0.79-2.01); *P* = .34) (eTable 3 in the Supplement). The overall χ^2^ test did not show significant differences among propensity score coefficients (*P* = .61).

Descriptive statistics of clinical characteristics of patients in the propensity score–matched sample, according to the presence or absence of vertebral fracture, are reported in eTable 4 in the Supplement. The univariate logistic regression analysis, using the complete set of data, demonstrated a significant association between vertebral fracture prevalence and age (OR, 1.08; 95% CI, 1.04-1.12; *P* < .001), previous fractures (OR, 3.04; 95% CI, 1.75-5.45; *P* < .001), total hip BMD (OR, 0.08; 95% CI, 0.01-0.95; *P* = .046), and total hip *T* score (OR, 0.73; 95% CI, 0.53-0.99); *P* = .04) (eTable 4 in the Supplement).

The aforementioned covariates (age, previous fractures, total hip BMD, total hip *T* score), *ERRB2* status, and the multiplicative interaction term between AI group and FBM (after transformation of FBM values in kilograms to natural logarithms) were entered in the multivariable analysis, which confirmed a statistically significant association of age, previous fractures, and interaction term with the vertebral fracture prevalence (eTable 5 in the Supplement). In particular, a 1-year increase in age was associated with 8% increased odds of having a morphometric vertebral fracture, maintaining constant the other covariates (OR, 1.08; 95% CI, 1.04-1.12; *P* < .001). Using patients without previous fractures as the reference, the odds of having a vertebral fracture were approximately 3 times more likely in patients with previous fractures (OR, 2.96; 95% CI, 1.67-5.25; *P* < .001). With regard to the multiplicative interaction term, the association of FBM with vertebral fracture prevalence was significantly different according to the AI group (OR for the interaction, 5.77; 95% CI, 1.08-30.81; *P* for interaction term = .03 [eTable 5 in the Supplement]). The stratification analysis (Table 3) showed that a 1-unit increase in the natural logarithm of FBM produced a numerical 94% increased odds of having a vertebral fracture in the AI-treated patients, whereas a numerical 62% reduced odds of vertebral fracture was observed for a 1-unit FBM increase in the AI-naive patients (OR, 1.94 [95% CI, 0.67-5.64] and 0.38 [95% CI, 0.12-1.19], respectively).

**Table 3.** Stratification Analysis by AI Group on the Chance of Vertebral Fracture With Covariate Adjustment After Propensity Score Matching Among 374 Patients With Early Breast Cancer

Characteristic	AI-Naive Group (n = 187)			AI-Treated Group (n = 187)		
	Morphometric Vertebral Fracture		OR (95% CI)	Morphometric Vertebral Fracture		OR (95% CI)
	Absence (n = 146 [78.1%])	Presence (n = 41 [21.9%])		Absence (n = 134 [71.7%])	Presence (n = 53 [28.3%])	
Age, mean (SD), y	64.9 (8.3)	69.4 (6.5)	1.09 (1.04-1.16)	65.2 (7.5)	68.7 (7.4)	1.07 (1.02-1.12)
Previous fractures, No. (%)						
No	118 (81.9)	26 (18.01)	1 [Reference]	107 (78.7)	29 (21.3)	1 [Reference]
Yes	28 (65.1)	15 (34.9)	2.83 (1.26-6.33)	27 (52.9)	24 (47.1)	2.97 (1.45-6.11)
Fat body mass, mean (SD)^a^	3.21 (0.31)	3.12 (0.36)	0.38 (0.12-1.19)	3.21 (0.34)	3.31 (0.30)	1.94 (0.67-5.64)

Abbreviations: AI, aromatase inhibitor; NA, not applicable; OR, odds ratio.

^a^ Fat body mass was analyzed as the natural logarithm values.

**Figure 2.**
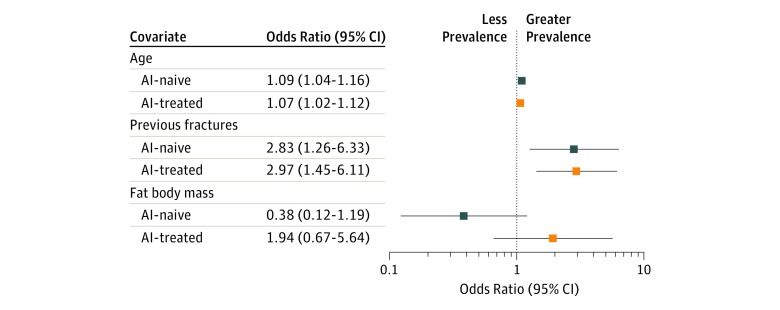
Prevalence Odds Ratios for Vertebral Fracture Associated With Age, Previous Fractures, and Fat Body Mass, by Aromatase Inhibitor (AI) Group in the Propensity Score–Matched Sample Error bars for age are not perceptible because of small 95% CIs.

The stratification analysis (Table 3) showed that a 1-unit increase in FBM produced 94% increased odds of having a vertebral fracture in the AI-treated patients, whereas 62% reduced odds of vertebral fracture were observed for a 1-unit FBM increase in the AI-naive patients (OR, 1.94 [95% CI, 0.67-5.64] and 0.38 [95% CI, 0.12-1.19], respectively). A forest plot of the stratification analysis is presented in Figure 2.

### Exploratory Correlation Analyses Between TBS and FBM

We carried out TBS analysis in 128 of the 556 patients (23.0%): 78 in the AI-naive group and 50 in the AI-treated group. These women were consecutively included during the last 15 months of the study when the software for TBS analysis became available at our institution. The characteristics of the patients with TBS assessment as compared with the entire study population are listed in eTable 6 and eTable 7 in the Supplement. Trabecular bone score was partially degraded in both groups: mean TBS was 1.26 (95% CI, 1.24-1.29) in the AI-naive group and 1.22 (95% CI, 1.19-1.25) in the AI-treated group (*P* = .006) (Table 2). We observed an inverse correlation between FBM and TBS in both the AI-naive patients and the AI-treated patients (AI naive: Pearson *r* = –0.265; *P* = .02; AI treated: Pearson *r* = –0.406; *P* = .004) (eFigure 1 in the Supplement).

After propensity score matching (n = 86), the mean (SD) TBS was similar in the AI-naive and AI-treated groups (1.22 [0.10] vs 1.21 [0.10]). The univariate analysis showed a significant association between FBM and TBS: a 1-unit increase in the natural logarithm of FBM produced a –0.01 decrease in the mean TBS (β = –0.01; 95% CI, –0.02 to –0.01; *P* = .004). No significant association between AI group and TBS was found (β = 0.0031; 95% CI, –0.0376 to 0.0438; *P* = .88). The association of FBM with TBS was not significantly different according to the AI group (*P* for interaction term = .56).

## Discussion

This cross-sectional study reports a significant association between AI therapy and increased prevalence of vertebral fractures in postmenopausal women with early-stage breast cancer. As previously shown,^12^ low BMD was not associated with the occurrence of vertebral fractures in AI-treated patients.

The main finding of the present analysis is the observation of a difference in the association between vertebral fracture prevalence and AI therapy depending on FBM measurement by DXA. We previously reported that higher FBM was significantly associated with an increased risk of fracture in patients with prostate cancer undergoing androgen-deprivation therapy.^33^ The proportion of vertebral fractures was numerically lower in the AI-naive patients with above-the-median FBM than in those with low FBM, although the difference was not statistically significant. This suggests a protective role of adiposity against skeletal fragility resulting from the higher estrogen levels in obese women^34^ and the consequent increase in BMD. An opposite association of FBM with vertebral fracture prevalence was noted in the AI-treated patients. This observation is in line with the so-called obesity paradox: as mentioned previously, body fat can play a harmful role via several mechanisms in the development of bone fragility (eFigure 2 in the Supplement).^21^

These findings have clinical relevance since vertebral fractures are associated with an increased risk of new vertebral^35^ and other nonspinal fractures,^36^ chronic back pain,^37^ reduced quality of life,^38^ and, possibly, increased mortality in patients with osteoporosis.^39^ However, approximately one-third of vertebral fractures are clinically recognized.^40^ To assess the prevalence of vertebral fractures, we performed a morphometric analysis of DXA images, which has emerged as an accurate and reproducible method tested and applied in many clinical studies.^41^

The TBS is a US Food and Drug Administration–approved measure of bone texture generated from spinal BMD scans, and it is associated with bone microarchitecture. Cross-sectional studies have shown that greater TBS is inversely associated with risk of osteoporotic fractures in postmenopausal women and men,^29,30,42^ regardless of whether the BMD *T* score falls in the normal to osteopenic or osteoporotic range. In our study, FBM was inversely correlated with TBS in a subset of AI-naive and AI-treated patients, thus supporting the hypothesis of a role of FBM in disrupting bone quality in both these groups.

Older age and history of recent osteoporotic fractures are the most relevant predictors of fracture risk in the general population.^13,43^ In the present study, these clinical parameters were independently associated with a greater proportion of vertebral fractures in both the AI-naive and AI-treated patients. We noted that after adjusting for age and previous fractures, FBM maintained its protective role among the AI-naive patients but was associated with increased prevalence of vertebral fractures among the AI-treated patients. This divergence was statistically significant at the interaction test after propensity score matching. To our knowledge, the interaction of endocrine therapy with FBM on vertebral fracture prevalence in patients with early-stage breast cancer has not been described so far.

Noteworthy in our study, the increased proportion of vertebral fractures in the AI-treated patients, as opposed to the AI-naive ones, was confined to the subset with high FBM, whereas no difference in fracture prevalence according to AI therapy status was observed in patients with low FBM. These findings further underline the importance of FBM as a factor potentially associated with the skeletal morbidity of AIs.

This study suggests that obesity, which is associated with a reduced fracture risk in healthy postmenopausal women, might have the opposite association in patients with early breast cancer undergoing AI therapy. A plausible mechanism for this difference is that AIs inhibit the production of estrogen, leading to a loss of their protection against fragility-related fracture (eFigure 2 in the Supplement).

### Limitations

This study has several limitations. The cross-sectional design is the major limitation. Among the AI-treated participants, the proportion of vertebral fractures already present before initiating AI therapy is unknown. Moreover, because patients entered the study at different times during exposure to AIs, we were unable to determine whether obesity favored an earlier occurrence of fractures or not. It should be noted, however, that a previous analysis observed no significant increased prevalence of morphometric vertebral fractures with long-term AI therapy.^12^

In the AI-treated patients, the greater proportion of vertebral fractures associated with elevated FBM values was most evident with mild fractures (21.4% vs 9.3%), whereas the difference in terms of moderate or severe fractures between patients with high and low FBM was less evident (12.2% vs 11.3%). The clinical significance of mild (imaging-defined) vertebral fractures is uncertain, because they have heterogeneous underlying causes, being a consequence of either degenerative or early osteoporotic changes.^44^ Nonetheless, mild morphometric vertebral fractures were found to predict future vertebral fractures in a previous study^45^ and may have an association with future physical functioning,^46^ so their association with patient outcome is currently under investigation. Certainly, moderate to severe fractures have a greater clinical relevance because they are associated with future vertebral and nonvertebral fractures^45^ and reduced quality of life.^38^ The total number of moderate to severe vertebral fractures in our study, however, was too small to show any significant association with FBM, and this is a limitation. This study suggests that FBM may be associated with increased skeletal fragility in postmenopausal women undergoing AI therapy as demonstrated by a direct inverse association between FBM and TBS and the increased proportion of mild vertebral fractures in patients with higher FBM. These results are therefore exploratory and need confirmation. A prospective study by our group (ClinicalTrials.gov identifier NCT02166281), for which recruitment is nearly complete, could provide additional information in this respect.

## Conclusions

We suggest for the first time, to our knowledge, that FBM may be associated with fragility fractures in patients with breast cancer who are undergoing therapy with AIs. If these data are confirmed, obesity may become an additional parameter to be considered in the clinical decision of prescribing bisphosphonates or denosumab to reduce fracture risk in women who are candidates for endocrine therapy with an AI.

## Notes

Supplement. **eTable 1.** Distribution of Skeletal and Body Composition Parameters in Breast Cancer Patients With and Without Vertebral Fracture Stratified According to Whether They Underwent AI Therapy (AI-Treated) or Not (AI-Naive) **eTable 2.** Prevalence of Mild and Moderate/Severe Vertebral Fractures in AI-Naive and AI-Treated Patients by FBM Category (≥Median vs <Median Value) **eTable 3.** Demographic and Clinical Characteristics of AI-Naive and AI-Treated Patients Before (N = 483) and After Propensity Score Matching (N = 374) **eTable 4.** Contingency Tables and Output of the Univariate Analysis With Propensity Score Nearest Neighbor Matching (N = 374) **eTable 5.** Multivariable Analysis of Factors Potentially Associated With Vertebral Fracture Prevalence in the Propensity Score Matched Sample (N = 374) **eTable 6.** Clinical and Tumor Characteristics of Patients With TBS Assessment as Compared With the Entire Study Population **eTable 7.** Distribution of Dual-Energy X-ray Absorptiometry Parameters in Patients With TBS Assessment as Compared With the Entire Study Population **eFigure 1.** Pearson’s Correlation Between Trabecular Bone Score (TBS) and Fat Body Mass (in Grams) in the Aromatase Inhibitor (AI)-Naive (A) and the AI-Treated Group (B) **eFigure 2.** Diagrams of the Association Between Increased Fat Body Mass (FBM) and Vertebral Fractures
