# Supplementary material for: Association of Fat Body Mass With Vertebral Fractures in Postmenopausal Women With Early Breast Cancer Undergoing Adjuvant Aromatase Inhibitor Therapy
Source: JAMA Netw Open. 2019 Sep 27;2(9):e1911080. doi: 10.1001/jamanetworkopen.2019.11080 (PMC6777242; doi:10.1001/jamanetworkopen.2019.11080)
Supplement: Supplement. — eTable 1. Distribution of Skeletal and Body Composition Parameters in Breast Cancer Patients With and Without Vertebral Fracture Stratified According to Whether They Underwent AI Therapy (AI-Treated) or Not (AI-Naive) eTable 2. Prevalence of Mild and Moderate/Severe Vertebral Fractures in AI-Naive and AI-Treated Patients by FBM Category (≥Median vs <Median Value) eTable 3. Demographic and Clinical Characteristics of AI-Naive and AI-Treated Patients Before (N = 483) and After Propensity Score Matching (N = 374) eTable 4. Contingency Tables and Output of the Univariate Analysis With Propensity Score Nearest Neighbor Matching (N = 374) eTable 5. Multivariable Analysis of Factors Potentially Associated With Vertebral Fracture Prevalence in the Propensity Score Matched Sample (N = 374) eTable 6. Clinical and Tumor Characteristics of Patients With TBS Assessment as Compared With the Entire Study Population eTable 7. Distribution of Dual-Energy X-ray Absorptiometry Parameters in Patients With TBS Assessment as Compared With the Entire Study Population eFigure 1. Pearson’s Correlation Between Trabecular Bone Score (TBS) and Fat Body Mass (in Grams) in the Aromatase Inhibitor (AI)-Naive (A) and the AI-Treated Group (B) eFigure 2. Diagrams of the Association Between Increased Fat Body Mass (FBM) and Vertebral Fractures [file jamanetwopen-2-e1911080-s001.pdf]

Supplementary Online Content

Pedersini R, Amoroso V, Maffezzoni F, et al. Association of fat body mass with vertebral fractures in postmenopausal women with early breast cancer undergoing adjuvant aromatase inhibitor therapy. *JAMA Netw Open*. 2019;2(9):e1911080. doi:10.1001/jamanetworkopen.2019.11080

eTable 1. Distribution of Skeletal and Body Composition Parameters in Breast Cancer Patients With and Without Vertebral Fracture Stratified According to Whether They Underwent AI Therapy (AI-Treated) or Not (AI-Naive)

eTable 2. Prevalence of Mild and Moderate/Severe Vertebral Fractures in AI-Naive and AI-Treated Patients by FBM Category (≥Median vs <Median Value)

eTable 3. Demographic and Clinical Characteristics of AI-Naive and AI-Treated Patients Before (N = 483) and After Propensity Score Matching (N = 374)

eTable 4. Contingency Tables and Output of the Univariate Analysis With Propensity Score Nearest Neighbor Matching (N = 374)

eTable 5. Multivariable Analysis of Factors Potentially Associated With Vertebral Fracture Prevalence in the Propensity Score Matched Sample (N = 374)

eTable 6. Clinical and Tumor Characteristics of Patients With TBS Assessment as Compared With the Entire Study Population

eTable 7. Distribution of Dual-Energy X-ray Absorptiometry Parameters in Patients With TBS Assessment as Compared With the Entire Study Population

eFigure 1. Pearson's Correlation Between Trabecular Bone Score (TBS) and Fat Body Mass (in Grams) in the Aromatase Inhibitor (AI)-Naive (A) and the AI-Treated Group (B)

eFigure 2. Diagrams of the Association Between Increased Fat Body Mass (FBM) and Vertebral Fractures

This supplementary material has been provided by the authors to give readers additional information about their work.

eTable 1. Distribution of skeletal and body composition parameters in breast cancer patients with and without vertebral fracture stratified according to whether they underwent AI therapy (AI-treated) or not (AI-naïve).

Parameter (mean, 95% CI)	AI-naïve patients (N = 361)		*P* value*	AI-treated patients (N = 195)		*P* value*
	No fracture (n = 302)	Fracture (n = 59)		No fracture (n = 142)	Fracture (n = 53)	
Lumbar spine BMD, g/cm^2	0.90 (0.88-0.92)	0.87 (0.82-0.91)	0.10	0.86 (0.84-0.88)	0.88 (0.84-0.92)	0.72
Femoral neck BMD, g/cm^2	0.70 (0.69-0.71)	0.65 (0.63-0.68)	0.002	0.68 (0.66-0.69)	0.69 (0.66-0.72)	0.55
Total hip BMD. g/cm^2	0.82 (0.81-0.84)	0.77 (0.74-0.79)	0.001	0.81 (0.79-0.82)	0.82 (0.78-0.84)	0.84
Lumbar spine T-score	-1.22 (-1.37- -1.05)	-1.31 (-1.77- -0.83)	0.41	-1.58 (-1.75- -1.40)	-1.51 (-1.86- -1.15)	0.97
Femoral neck T-score	-1.33 (-1.4- -1.2)	-1.77 (-1.9- -1.52)	0.002	-1.51 (-1.61- -1.38)	-1.38 (-1.66- -1.10)	0.54
Total hip T-score	-0.91 (-1- -0.8)	-1.39 (-1.6- -1.1)	0.001	-1.08 (-1.20- -0.96)	-1.04 (-1.29- -0.78)	0.76
Fat body mass, grams	24,911 (23,972-25,850)	23,951 (21,667-26,234)	0.21	25,915 (24,407-27,422)	28,717 (26,343-31,092)	0.03

Abbreviations: AI, aromatase inhibitor; BMD, bone mineral density; BMI, body mass index; CI, confidence interval.

**P* value from the Mann-Whitney U test.

eTable 2. Prevalence of mild and moderate/severe vertebral fractures in AI-naïve and AI-treated patients by FBM category (≥ median vs. < median value).

	Low FBM group (< median)		High FBM group (≥ median)	
	**AI-naïve patients (N = 180)**	**AI-treated patients (N = 97)**	**AI-naïve patients (N = 181)**	**AI-treated patients (N = 98)**
**Vertebral fractures, No. (%)**				
No fracture	145 (80.6)	77 (79.4)	157 (86.7)	65 (66.3)
Mild fractures	15 (8.3)	9 (9.3)	16 (8.8)	21 (21.4)
Moderate/severe fractures	20 (11.1)	11 (11.3)	8 (4.4)	12 (12.2)
P* value	0.96		< 0.001	

Abbreviations: AI, aromatase inhibitor; FBM, fat body mass.

**P* value from the chi-square test.

eTable 3. Demographic and clinical characteristics of AI-naïve and AI-treated patients before (N = 483) and after propensity score matching (N = 374).

	Before matching				After matching	
Characteristic, No. (%) or mean (SD)	Descriptive statistic		Univariate analysis		Univariate analysis	
	AI Group		OR (95% CI)	*P* value*	OR (95% CI)	*P* value*
	AI-naïve (296 (61.3)	AI-treated (187 (38.7)				
**Age, years**	61.33 (10.1)	66.07 (7.7)	1.06 (1.04 - 1.08)	<0.001	1.00 (0.98 - 1.03)	0.74
**BMI, kg/m^2**	25.34 (4.7)	26.36 (4.6)	1.05 (1.01 - 1.09)	0.02	1.03 (0.99 - 1.08)	0.19
**Ki-67 index, %**	25.0 (16.4)	18.4 (15.2)	0.97 (0.96 - 0.98)	<0.001	1.00 (0.98 - 1.01)	0.66
**HER2 status**				0.006		0.87
Negative	280 (62.5)	168 (37.5)	1		1	
Positive	81 (76.4)	25 (23.6)	0.51 (0.31 - 0.83)		1.05 (0.56 - 1.97)	
**Previous Fractures**				0.002		0.34
No	295 (67.7)	141 (32.3)	1		1	
Yes	55 (51.4)	52 (48.6)	1.98 (1.29 - 3.04)		1.26 (0.79 - 2.01)	
**Physical activity**				0.05		0.12
No	221 (63.7)	126 (36.3)	1		1	
Yes	79 (54.1)	67 (45.9)	1.49 (1 - 2.2)		1.42 (0.91 - 2.20)	
**Smoking status**				0.86		
No	281 (63.6)	161 (36.4)	1			
Yes	60 (64.5)	33 (35.5)	0.96 (0.6 - 1.52)			
**Alcohol consumption**				0.002		0.06
No	258 (66.1)	132 (33.9)	1		1	
Yes	61 (50)	61 (50)	1.95 (1.29 - 2.96)		1.54 (0.97 - 2.43)	
**Chemotherapy use**				<0.001		0.91

No	200 (59.2)	138 (40.8)	1		1	
Yes	161 (74.5)	55 (25.5)	0.5 (0.34 - 0.72)		0.97 (0.62 - 1.53)	

Abbreviations: AI, aromatase inhibitor; BMI, body mass index; CI, confidence interval; OR, odds ratio.

*Likelihood Ratio *P*-value.

eTable 4. Contingency tables and output of the univariate analysis with propensity score nearest neighbor matching (N = 374).

Characteristic, No. (%) or mean (SD)	Descriptive statistic		Univariate analysis	
	Morphometric vertebral fracture		OR (95% CI)	P value**
	**Absence** (280 (74.9)	**Presence** (94 (25.1)		
**AI group**				0.13
AI-naïve	146 (78.1)	41 (21.9)	1	
AI-treated	134 (71.7)	53 (28.3)	1.47 (0.89 - 2.46)	
**Age *, years**	65.0 (7.9)	69.0 (7.0)	1.08 (1.04 - 1.12)	<0.001
**BMI, kg/m^2**	26.13 (4.6)	26.39 (4.7)	1.01 (0.96 - 1.07)	0.69
**Smoking status**				0.72
No	226 (74.3)	78 (25.7)	1	
Yes	54 (77.1)	16 (22.9)	0.88 (0.44 - 1.74)	
**Alcohol consumption**				0.47
No	205 (75.9)	65 (24.1)	1	
Yes	75 (72.1)	29 (27.9)	1.24 (0.68 - 2.24)	
**Physical activity**				0.74
No	190 (74.2)	66 (25.8)	1	
Yes	90 (76.3)	28 (23.7)	0.91 (0.51 - 1.60)	
**Pathologic tumor stage**				0.40
pT1	194 (74.3)	67 (25.7)	1	
pT2	76 (75.2)	25 (24.8)	0.95 (0.52 - 1.70)	
pT3-4	10 (90.9)	1 (9.1)	0.27 (0.01 - 1.68)	
**Pathologic nodal status**				0.33
pN0	168 (72.7)	63 (27.3)	1	
pN1	93 (79.5)	24 (20.5)	0.64 (0.34 - 1.15)	
pN2-3	16 (76.2)	5 (23.8)	0.84 (0.24 - 2.58)	
**Ki-67 index, %**	19.17 (14.2)	17.24 (10.9)	0.99 (0.97 - 1.01)	0.28
**HER2 status**				0.05
Negative	241 (73.2)	88 (26.8)	1	
Positive	39 (86.7)	6 (13.3)	0.41 (0.14 - 1.01)	
**Chemotherapy use**				0.17
No	194 (72.7)	73 (27.3)	1	
Yes	86 (80.4)	21 (19.6)	0.66 (0.35 - 1.2)	

**Previous Fractures ***				<0.001
No	225 (80.3)	55 (19.6)	1	
Yes	55 (58.5)	39 (41.5)	3.04 (1.75 - 5.45)	
**Lumbar spine BMD, g/cm^2**	0.88 (0.13)	0.87 (0.15)	0.79 (0.11 - 5.31)	0.81
**Lumbar spine T-score**	-1.45 (1.25)	-1.43 (1.51)	1.01 (0.82 - 1.22)	0.95
**Femoral neck BMD, g/cm^2**	0.69 (0.1)	0.67 (0.11)	0.31 (0.02 - 4.49)	0.38
**Femoral neck T-score**	-1.44 (0.84)	-1.57 (0.97)	0.85 (0.63 - 1.15)	0.29
**Total hip BMD *, g/cm^2**	0.82 (0.1)	0.79 (0.11)	0.08 (0.01 - 0.95)	0.05
**Total hip T-score ***	-1 (0.83)	-1.22 (0.91)	0.73 (0.53 - 0.99)	0.04
**Lean body mass, kg**	3.68 (0.12)	3.68 (0.13)	0.79 (0.09 - 6.62)	0.83
**Fat body mass, kg**	3.21 (0.32)	3.23 (0.34)	1.2 (0.54 - 2.69)	0.65

Abbreviations: AI, aromatase inhibitor; BMD, bone mineral density; BMI, body mass index; CI, confidence interval; OR, odds ratio.

*Variables entering the multivariable analysis.

**Likelihood Ratio *P*-value.

eTable 5. Multivariable analysis of factors potentially associated with vertebral fracture prevalence in the propensity score matched sample (N = 374).

**Characteristic**	**Multivariable analysis**	
	**OR (95% CI)**	***P* value***
(Intercept)	0.03 (0.01 - 2.16)	0.11
**Age, years**	1.08 (1.04 - 1.12)	<0.001
**Previous Fractures**		<0.001
No	1	
Yes	2.96 (1.67 - 5.25)	
**AI group**		0.05
AI-naïve	1	
AI-treated	0.01 (0.00 - 1.09)	
**Fat Body Mass**		0.10
< median		
≥ median	0.36 (0.11 - 1.21)	
**AI group*Fat Body Mass**		0.03
AI-naïve : Fat Body Mass	1	
AI-treated : Fat Body Mass	5.77 (1.08 - 30.81)	

Abbreviations: AI, aromatase inhibitor; CI, confidence interval; OR, odds ratio.

*Likelihood Ratio *P*-value.

eTable 6. Clinical and tumor characteristics of patients with TBS assessment as compared with the entire study population.

**Characteristic (No., %)**	**Patients with TBS assessment (N = 128)**	**All patients (N = 556)**	***P* value***
**Age, years (mean, 95% CI)**	63.1 (61.1-65.0)	63.0 (62.2-63.8)	0.44
**BMI, kg/m^2 (mean, 95% CI)**	25.6 (24.7-26.3)	25.55 (24.8-26.3)	0.89
**Pathologic tumor stage**			0.88
pT1	89 (70.6)	380 (68.8)	
pT2	33(26.2)	153 (27.7)	
pT3-4	4 (3.2)	20 (3.6)	
**Pathologic nodal stage**			0.69
pN0	78 (60.9)	335 (60.2)	
pN1	42 (32.8)	183 (32.9)	
pN2-3	5 (3.9)	6 (6.5)	
Unknown	3 (2.3)	2 (0.4)	
**Ki-67 index (mean, 95% CI)**	22.7 (21-24)	23.7 (21-26)	0.09
**HER2 status**			0.12
Negative	104 (81.3)	448 (80.9)	
Positive	24 (18.7)	106 (19.1)	
**Chemotherapy**			0.35
No	84 (65.6)	373 (67.3)	
Yes	44 (34.4)	181 (32.7)	
**Physical activity**			0.009
No	96 (82.1)	358 (71.0)	
Yes	21 (17.9)	146 (29.0)	
**Alcohol consumption**			0.02
No	103 (85.1)	397(76.5)	
Yes	18 (14.9)	122 (23.3)	
**Smoking status**			0.01
No	110 (90.9)	449 (82.8)	
Yes	11 (9.1)	93 (17.2)	

**Previous fractures**			0.005
No	107 (83.6)	436 (78.4)	
Yes	13 (10.2)	107 (19.2)	
Unknown	8 (6.3)	13 (2.4)	

Abbreviations: AI, aromatase inhibitor; BMD, bone mineral density; BMI, body mass index; CI, confidence interval.

*P value from the Mann-Whitney U test or the chi-square test as appropriate.

eTable 7. Distribution of dual-energy x-ray absorptiometry parameters in patients with TBS assessment as compared with the entire study population.

Characteristic (No., %)	Patients with TBS assessment (N = 128)	All patients (N = 556)	*P* value*
**DXA**			0.51
Normal	26 (20.3)	103 (18.5)	
Osteopenia/Osteoporosis	102 (79.7)	453 (81.5)	
**Morphometric vertebral fracture**			0.20
Not present	107 (83.6)	444 (79.9)	
Present	21 (16.4)	107 (83.6)	
**Vertebral fracture grade**			0.03
No	107 (83.6)	444 (79.9)	
Mild	5 (3.9)	62 (11)	
Moderate/severe	16 (12.5)	51 (9.2)	
**Lumbar spine BMD, g/cm^2 (mean, 95% CI)**	0.91 (0.88-0.93)	0.88 (0.87-0.90)	0.09
**Lumbar spine T-score (mean, 95% CI)**	-1.22 (-1.45-1.98)	-1.34 (-1.45- -1.22)	0.19
**Femoral neck BMD, g/cm^2 (mean, 95% CI)**	0.69 (0.67-0.71)	0.69 (0.68-0.69)	0.98
**Femoral neck T-score (mean, 95% CI)**	-1.43 (-1.59- -1.27)	-1.42 (-1.50- -1.35)	0.98
**Total hip BMD, g/cm^2 (mean, 95% CI)**	0.82 (0.80-0.84)	0.81 (0.80-0.82)	0.43
**Total hip T-score (mean, 95% CI)**	-0.98 (-1.13- -0.82)	-1.02 (-1.09- -0.94)	0.38
**Lean body mass, grams (mean, 95% CI)**	41,227.5 (40,313.6 - 42,141.5)	39,712.7 (39,295.4 - 40,130.1)	0.002
**Fat body mass, grams (mean, 95% CI)**	24,118.4 (23,696.6 - 26,540.3)	26,439.6 (24,265.0 - 28,614.2)	0.19

Abbreviations: AI, aromatase inhibitor; BMD, bone mineral density; CI, confidence interval; TBS, trabecular bone score.

**P* value from the Mann-Whitney U test or the chi-square test as appropriate.

eFigure 1. Pearson's correlation between trabecular bone score (TBS) and fat body mass (in grams) in the aromatase inhibitor (AI)-naïve (A) and the AI-treated group (B).

A

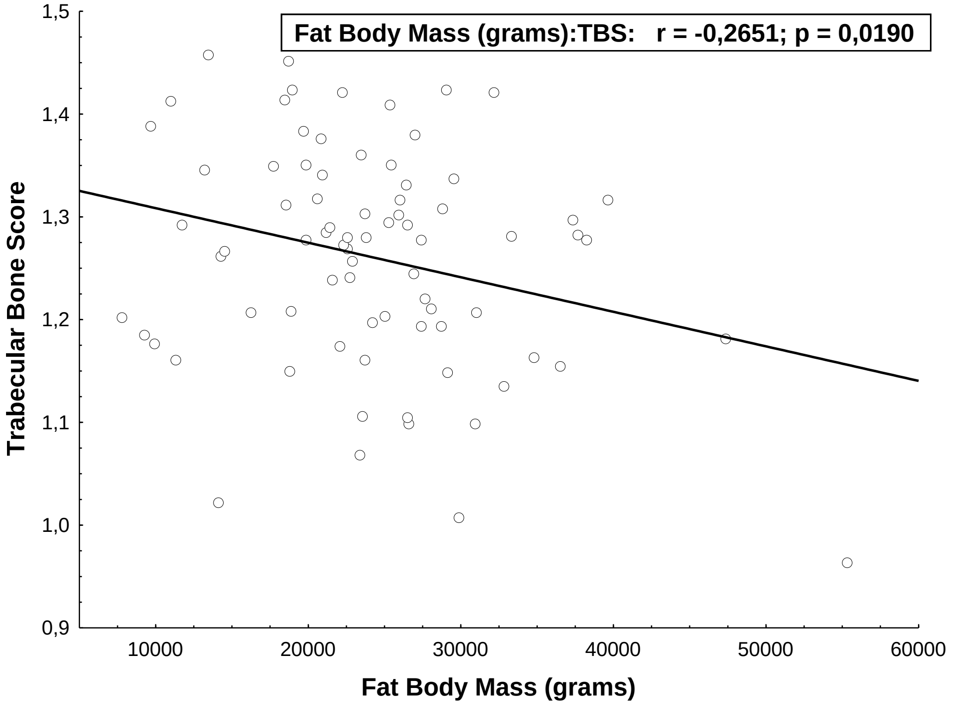

B

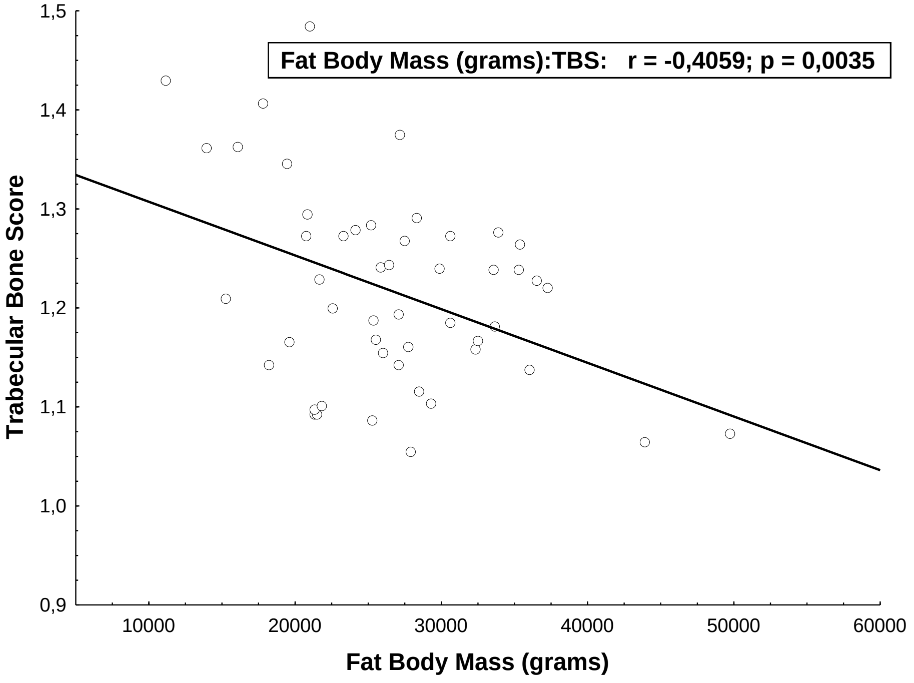

eFigure 2. Diagrams of the association between increased fat body mass (FBM) and vertebral fractures.

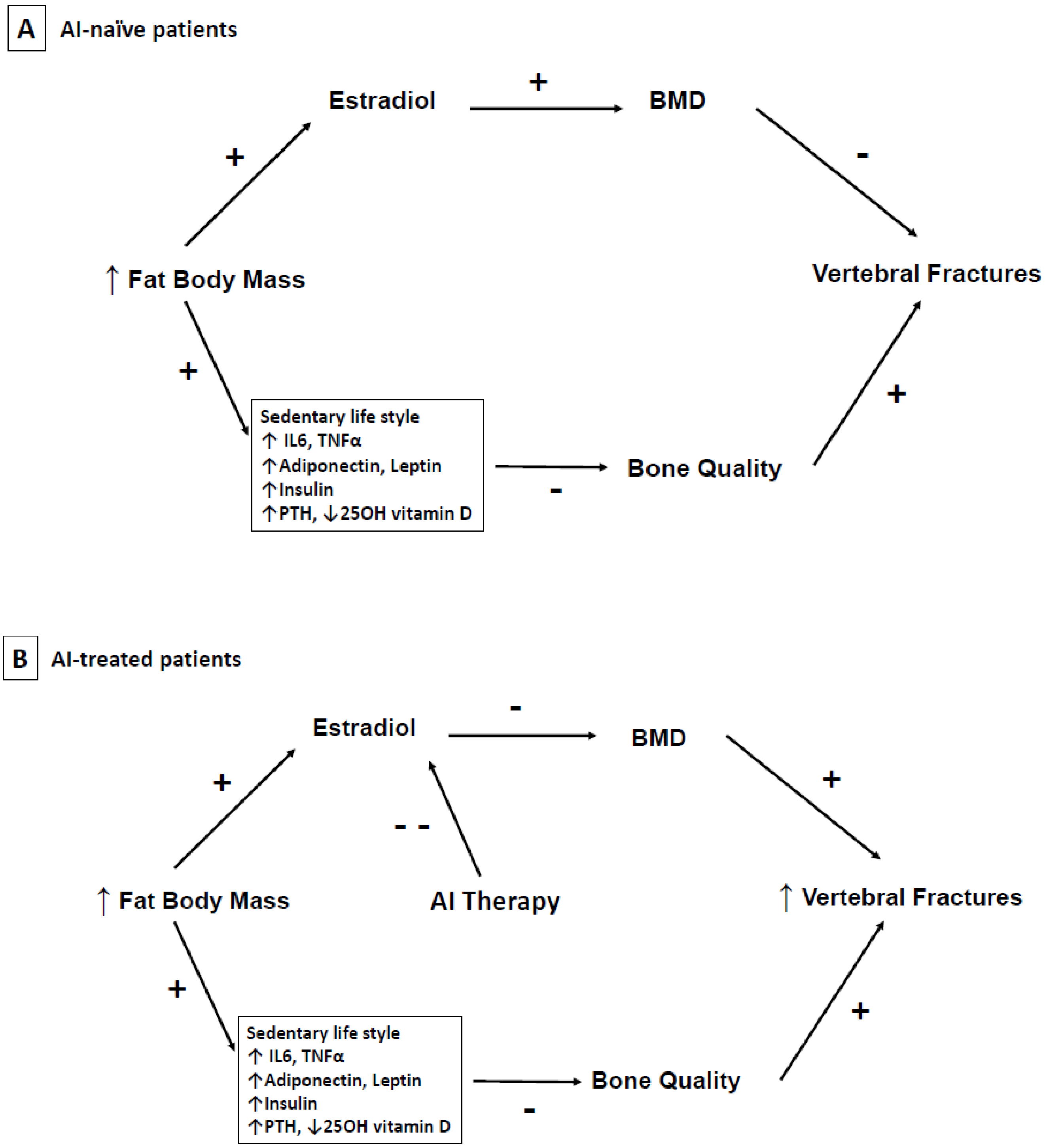

Legend: In the aromatase inhibitor (AI)-naïve patients (A), increased FBM is associated with bone quality deterioration due to increased inflammation proteins such as interleukin 6 (IL6), tumor necrosis factor α (TNFα),

increased levels of adiponectin and leptin, insulin and parathyroid hormone (PTH), and decreased levels of 25-OH vitamin D. This negative effect is compensated by higher bone mineral density due to elevated estrogen levels as a consequence of aromatase activity. In the AI-treated patients (B), the loss of estrogens due to aromatase inhibition results in lower BMD and this contributes to increase the fracture risk favored by bone quality deterioration.

+, positive effect, - negative effect.
